# Dynamic of competitive Lotka-Volterra model for tumor-host systems under constant or periodic perturbation: Implications for the therapy of cancer

**DOI:** 10.1371/journal.pone.0329087

**Published:** 2025-08-25

**Authors:** Rolando Placeres Jimenez, Luis E. Bergues Cabrales, Juan I. Montijano

**Affiliations:** 1 Grupo de Simulaç ao Computacional, Departamento de Física, Universidade Federal de S ao Carlos, S ao Carlos, S ao Paulo, Brazil; 2 Departamento de Investigación e Innovación, Centro Nacional de Electromagnetismo Aplicado, Universidad de Oriente, Santiago de Cuba, Cuba; 3 Instituto Universitario de Investigación en Matemáticas y Aplicaciones, Universidad de Zaragoza, Zaragoza, Spain; Federal University of Technology - Parana, BRAZIL

## Abstract

In this paper, the tumor-host interaction is modeled using a Lotka-Volterra framework. The critical parameters that define the possible dynamical regimes are identified through linear stability analysis. The effects of both constant and periodic perturbations are examined, along with their clinical implications. The treatment dose required to drive the system to a desired state is determined. It is also shown that aggressive tumors evolve toward a limit cycle when the host is under the action of low-frequency periodic treatment. As the frequency increases, a transition to a non-chaotic attractor occurs. This transition narrows as the frequency of the external periodic perturbation increases. No chaotic behavior is observed, even at higher values of both perturbation strength and frequency, as the maximum Lyapunov exponent remains negative. These results suggest that although aggressive tumors may not be completely eradicated by conventional anticancer therapies, they could potentially be controlled through external low-frequency periodic treatments that target directly only the host, such as immunotherapy.

## Introduction

Cancer remains a leading cause of death worldwide, despite significant advances in molecular and cellular biology, medicine, and biophysics. The effectiveness of many anticancer therapies remains limited, and a complete cure for cancer has yet to be achieved [1]. Experimental anticancer therapies have largely remained empirical, based on trial and error [2,3]. Integrating mathematical modeling with experimental oncology is a necessary step toward a deeper understanding of cancer genesis, growth, progression, invasion, and metastasis, as well as for developing personalized anticancer therapies targeting the host, whether patients (in clinical studies) or laboratory animals (in preclinical studies) [2,3].

Mathematical models of population ecology provide a correct theoretical framework to describe the dynamics of interaction between malignant and normal cells [4–9]. Population models are a useful theoretical tool for understanding tumor-host dynamics under various anticancer therapies, including onco-specific treatments and therapies under investigation (e.g., immunotherapy, electrochemical ablation, irreversible electroporation).

Mathematical models from population ecology offer a suitable theoretical framework for describing the dynamics of interactions between malignant and normal cells [4–9]. Such models are valuable tools for understanding tumor-host dynamics under various anticancer therapies, including oncoespecific treatments and herapies under investigation (e.g., immunotherapy, electrochemical ablation, irreversible electroporation).

Population models can help improve the effectiveness of anticancer therapies. Among these, the Lotka-Volterra (LV) model has been widely and successfully used [4–9]. One of its distinctive features is that population growth is limited. limited. Moreover, the LV model allows for the analysis of the four types of tumor responses treated with oncoespecific therapies (e.g., radiotherapy).

Numerous studies have examined the impact of periodic coefficients and external perturbations within the LV model [10–12], motivated by the oscillatory behavior of the original LV model without the logistic term [13–15]. The competitive LV model has been successfully applied to various problems in Ecology [16–23]. Cushing [20,21] and Gopalsamy [22] demonstrated that two competing species influenced by external periodic perturbations can coexist, without one being eliminated by the other, avoiding extinction. Similar behavior has been observed in immune system models under periodic perturbations [27–29], prompting researchers to propose such perturbations as potential therapeutic strategies to control cancer [28].

In experimental oncology, both internal (endogenous) and external (exogenous) perturbations affecting the host can alter tumor-host dynamics, tumor growth kinetics, and the biological and electrical properties of both tissues. Most studies focus on tumor-directed therapies, yet it remains underexplored whether the host alone can govern tumor-host dynamics—either in the absence of external perturbations or when only the host is perturbed, without directly targeting the tumor. For this reason, both types of perturbations are addressed in separate sections of this study: “The model and linear stability analysis” and “About the existence of periodic orbits” sections examine the system without external perturbations, while “Cancer and host cell populations under an external perturbation” section focuses on the effects of external perturbations.

In the first section of this study, we review the dynamics of the LV model using linear stability analysis to determine the parameter sets that define each dynamical regime. We also prove the non-existence of periodic orbits, implying that the tumor-host system always converges to one of the equilibrium points: host extinction, tumor elimination, or coexistence of tumor and host cells at fixed levels. In the second section, we study the effects of external perturbations (representing cancer therapies) and discuss their clinical implications. Three types of therapy are analyzed: (1) continuous treatment with an effect proportional to cell populations, (2) continuous treatment with an effect independent of cell populations, and (3) periodic treatment with an effect independent of cell populations. To facilitate this analysis, the LV equations are decoupled, allowing the system’s dynamics to be interpreted as the motion of a particle in a force field. We then define the characteristic frequency of oscillation around stable fixed points. The dynamics of the perturbed system are examined through phase space portraits, and we determine the treatment dose required to shift the tumor-host system into different regimes. Additionally, the maximum Lyapunov exponent is computed for the periodically applied treatment to confirm the system’s non-chaotic behavior. In the final section, we summarize the main findings and present new insights that will be further developed in a future study.

## The model and linear stability analysis

We consider two interacting species competing for space and resources, i.e., the cancer cells population *x* and the host cells population *y*. The evolution of this competence between these two population types may be modelled by the LV equations [4–9], given by

$$\dot{x}=r_{x}x(1-\frac{x+\gamma y}{K})-\delta_{xy}xy,$$ (1)

$$\dot{y}=r_{y}y(1-\frac{x+\gamma y}{K})-\delta_{yx}yx,$$ (2)

where tumor and healthy cells grow according to a logistic model with growth rates *r*_*x*_ and *r*_*y*_, respectively. Both species have their growth limited by the presence of the other species. If tumor cells (in absence of healthy cells) have a carrying capacity *K*_*x*_ and the carrying capacity of healthy cells is *K*_*y*_, and $\gamma =K_{y}/K_{x}$, the system tumor-host will have a capacity bounded by the condition $x\;+\;\gamma y\leq K=K_{x}$. In absence of the other terms, the system will grow until x+γy=K is satisfied. The parameter $\delta_{xy}$ measures the cancer cells destroyed by the host and $\delta_{yx}$ measures the host cells destroyed by the cancer. The equations can be rewritten as

$$\dot{x}=r_{x}x(1-\frac{x}{K_{x}})-\alpha_{xy}xy,$$ (3)

$$\dot{y}=r_{y}y(1-\frac{y}{K_{y}})-\alpha_{yx}yx,$$ (4)

where the parameter $\alpha_{xy}=\delta_{xy}\;+\;r_{x}\gamma /K$ is a measure of the interaction established by the host cells population in the presence of cancer cells population and $\alpha_{yx}$ is a measure of the interaction established by cancer cells population with the host cells population. Note that since $\gamma =K_{y}/K_{x}$, then $r_{x}\gamma /K=r_{x}/K_{x}$, and the parameter $\alpha_{xy}$ must be lower bounded by $\alpha_{xy}\geq r_{x}/K_{x}$. Similarly, it must be $\alpha_{yx}\geq 1/K_{y}$. All these parameters are assumed as non-negative constants.

Using the following transformation of variables

$$\tau =r_{x}t,\;x=K_{x}u,\;y=\frac{r_{x}K_{y}}{r_{y}}v,$$ (5)

the Eqs (3) and (4) reduce to the form

$$\dot{u}=u-u^{2}-cuv,$$ (6)

$$\dot{v}=dv-v^{2}-fuv,$$ (7)

with

$$c=\frac{K_{y}\alpha_{xy}}{r_{y}},\;d=\frac{r_{y}}{r_{x}},\;f=\frac{K_{x}\alpha_{yx}}{r_{x}}.$$ (8)

In virtue of this transformation, the Eqs (6) and (7) are a LV model depending upon three dimensionless parameters. This formulation is very convenient because the transformed variables are dimensionless and reduces the number of parameters, which makes easier the analysis. Furthermore, the dimensionless form of equations is extensively used in many studies, play an important role serving as parameters in differential equations, and allows describing characteristics without dimension or explicit expression unit.

The system of ordinary differential Eqs (6) and (7) has four equilibrium points *P*_1_(0,0), *P*_2_(0,*d*), *P*_3_(1,0) and $P_{4}=\left(\frac{1-cd}{1-cf},\frac{d-f}{1-cf}\right)$. The eigenvalues of the Jacobian matrix for each of these points are given by

$$\begin{array}{c} P_{1}=(0,0)\to \lambda_{1}=1,\lambda_{2}=d,\\ P_{2}=(0,d)\to \lambda_{1}=1-cd,\lambda_{2}=-d,\\ P_{3}=(1,0)\to \lambda_{1}=1,\lambda_{2}=d-f,\\ P_{4}=\left(\frac{1-cd}{1-cf},\frac{d-f}{1-cd}\right)\to \lambda_{1,2}=\frac{1-cd\;+\;d-f}{2(cf-1)}\pm \frac{1}{2}\sqrt{{\left(\frac{1-cd\;+\;d-f}{cf-1}\right)}^{2}\;+\;\frac{4(1-cd)(d-f)}{cf-1}}. \end{array}$$ (9)

Note that in the original variables, the equilibrium points *P*_1_, *P*_2_, *P*_3_ and *P*_4_ are (0,0), (0,*K*_*x*_), (*K*_*y*_,0) and $\left(\frac{K_{x}r_{y}(r_{x}-K_{y}\alpha_{xy})}{r_{y}r_{x}-K_{y}K_{x}\alpha_{xy}\alpha_{yx}},\frac{r_{x}K_{y}(r_{y}-K_{x}\alpha_{yx})}{r_{y}r_{x}-K_{y}K_{x}\alpha_{xy}\alpha_{yx}}\right)$, respectively.

The equilibrium point *P*_1_ is an unstable node. There exists four different regimes that are described below.

**Fig 1:**
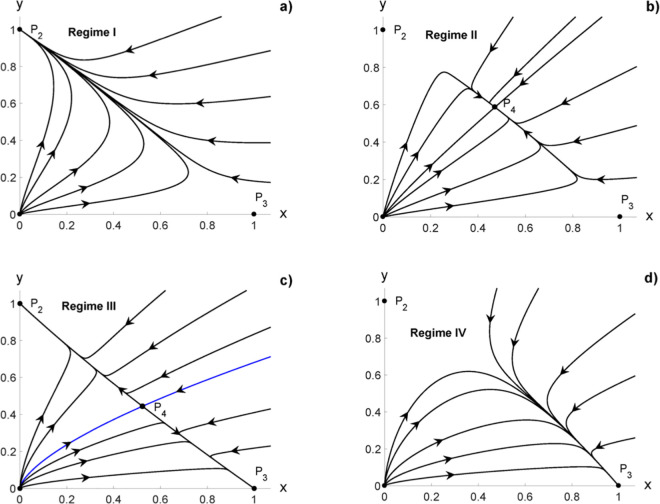
Phase portrait in the original variables: a) Regime I for cd > 1 and f < d. b) Regime II for *cf* < 1, *cd* < 1 and *f* < *d*. c) Regime III for *cf* > 1, *cd* > 1 and *f* > *d*. The separatrix in given in blue. d) Regime IV for *cd* < 1 and *f* > *d*.

- **Regime I:** *cd* > 1 and *f* < *d*
  The point *P*_2_ is a stable node, and *P*_3_ is a saddle point. In the phase portrait, all the trajectories converge toward the node *P*_2_ (Fig 1a). This case may correspond to one of the types of unperturbed tumor responses that may occur naturally without the action of any external perturbation (e.g., an external anticancer therapy), named natural tumor complete remission, which may be achieved by two possible ways. First, the cancer host alone is strong enough to eliminate any unperturbed cancer formed in a long finite time. This may suppose that the psico-neuro-endocrine-metabolic-immunological system of the host (host as a whole) is strong and stable. Second, the spontaneous remission of the unperturbed tumor (tumor appears and then dissapears in a relatively short finite time). This tumor spontaneous remission may be explained because the unperturbed tumor does not find adequate biophysical conditions (metabolic, electric and energetic) in the host for its formation and growth. The duration of these two ways depends on the tumor histological variety and host type. In clinics, the cancer patient behaves like an apparently healthy individual for both ways.
- The absence of external perturbations, as anticancer therapies, is refered to newly diagnosed cancer patients or who do not wish to receive none anticancer therapy of their own volition (Fig 1). The Fig 1a) is obtained using the original coordinates, taken as parameters $K_{x}=K_{y}=1$ cells, *r*_*x*_ = 0.1 days^−1^, *r*_*y*_ = 0.075 days^−1^, $\alpha_{xy}=0.1237$ cells^−1^days^−1^ and $\alpha_{yx}=0.045$ cells^−1^days^−1^. This gives *c* = 1.65, *d* = 0.75 and *f* = 0.45; therefore, cd=1.2374>1 and *f* < *d* that correspond to Regime I.
- **Regime II:** *cf* < 1, *cd* < 1 and *f* < *d*
  The points *P*_2_ and *P*_3_ are saddle points, and *P*_4_ is a stable node. In this case, the tumor and the host coexist in equilibrium; all the trajectories converge toward the node *P*_4_ (Fig 1b). This may correspond to other two types of unperturbed tumor responses that may also occur naturally without the action of external perturbations, named natural tumor partial response or natural tumor stationary partial response. This first response type may occur when the cancer grows slowly over time (long duration response for a finite time). The second response type may occur when this coexistence remains throughout the life of the cancer patient. Therefore, the cancer may behave as a controlled disease without the need for the application of external perturbation (e.g., anticancer therapies). The last has been observed in some cancer patients in clinical oncology with long survival who are not received any type of anticancer therapy, whether oncoespecific (surgery, chemotherapy or radiotherapy) or under study/trial (e.g., immunotherapy, electrochemical therapy, irreversible electroporation) [30].
  The Fig 1b) is obtained using the original coordinates, taken as parameters $K_{x}=K_{y}=1$ cells, *r*_*x*_ = 0.1 days^−1^, *r*_*y*_ = 0.09 days^−1^, $\alpha_{xy}=0.09$ cells^−1^ days^−1^ and $\alpha_{yx}=0.0788$ cells^−1^ days^−1^. This gives *c* = 1, *d* = 0.9 and *f* = 0.788; therefore, *cd* < 1 *f* < *d* and *cf* < 1 that correspond to Regime II.
- **Regime III:** *cf* > 1, *cd* > 1 and *f* > *d*
  The points *P*_2_ and *P*_3_ are stable nodes, and *P*_4_ is a saddle point. The phase portrait is divided in two basin of attraction (Fig 1c); any initial state located in the upper left-hand evolves toward the node *P*_2_, whereas any trajectory in the lower right-hand side converges to node *P*_3_.
  In Regime II, the point *P*_4_ represents a state in which the tumor and the host can coexist, but this state is unstable. This case may correspond to another type of unperturbed tumor response that may also occur naturally without the action of external perturbations, named natural tumor stable disease.
  The natural tumor stable disease depends on whether the endogeneous perturbation (weak or strong), either in the tumor and/or in the host, is favorable or unfavorable for the host. This coexistence is dominated by the host (node *P*_2_) when the endogeneous perturbation is favorable, whereas this coexistence is dominated by the cancer for unfavorable perturbation (node *P*_3_).
  In clinics, favorable endogeneous pertubation may be acceptance and positive thoughts about the disease, adequate nutrition, good performance status, among others. Unfavorable endogeneous perturbation may be stress; unpleasant news received by the cancer patient; unstabilized concomitant disease (e.g., diabetes); self-organization and dynamic transformation of the unperturbed tumor, and noise sources inherent in it and the host that occur in space-time that lead to the growth, progression, invasion and metastasis of the cancer, as well as its protection against the attack of the immune system and resistance to anticancer therapies [31,32]. Furthermore, this self-organization leads to dynamic depletion of the host over time (most likely evolution towards node *P*_3_).
  The Fig 1c) is obtained using the original coordinates, taken as parameters $K_{x}=K_{y}=1$ cells, *r*_*x*_ = 0.1 days^−1^, *r*_*y*_ = 0.065 days^−1^, $\alpha_{xy}=0.0247$ cells^−1^ days^−1^ and $\alpha_{yx}=0.069$ cells^−1^ days^−1^. This gives *c* = 1.65, *d* = 0.65 and *f* = 0.69; therefore, *cd* < 1, *f* > *d* and *cf* > 1 that correspond to Regime III. The blue line in the figure corresponds to the separatrix that divides the phase space into a region of points that converge to *P*_3_ and another region of points that converge to *P*_2_
- **Regime IV:** *cd* < 1 and *f* > *d*
  The points *P*_2_ and *P*_3_ are a saddle point and stable node, respectively. In clinics, it corresponds to another unperturbed tumor response that may also occur naturally without the action of external perturbation (e.g., anticancer therapies), named natural disease progression. In this case, an undesirable prognosis for the cancer patient due to his deplorable performance status (lower values of the Karnofvky index or higher value of the ECOG scale) and/or marked tumor activity (e.g., very aggressive and/or advanced-stage tumors), as shown in Fig 1d). Any initial state evolves toward node *P*_3_, i.e., the unperturbed tumor leads to energetic and metabolic depletions of the host over time that may lead to its body depauperation, which may bring about also the death of it.
  The Fig 1d) is obtained using the original coordinates, taken as parameters $K_{x}=K_{y}=1$ cells, *r*_*x*_ = 0.1 days^−1^, *r*_*y*_ = 0.066 days^−1^, $\alpha_{xy}=0.066$ cells^−1^ days^−1^ and $\alpha_{yx}=0.07$ cells^−1^ days^−1^. This gives *c* = 1, *d* = 0.66 and *f* = 0.7; therefore, *cd* < 1 and *f* > *d* that correspond to Regime IV.

From the analysis performed in this section, it is clear that there is not oscillating solution. The existence of periodic solutions is treated in “About the existence of periodic orbits” section of this study. Strictly speaking, the assumption of constant parameters in our model is only an approximation valid inside certain time scales. Many processes in the host and its environment are cyclic (e.g., concentration of nutrients and oxygen, elimination of metabolic byproducts). In addition, cancer cells may develop control mechanism to modify the environment [4–8], which yield to a modification of the parameters. In spite of this limitation, the model describes adequately the different dynamic regimes of tumor evolution.

An important merit of this model is that there is not unlimited growth, i.e., all the trajectories in the phase portrait are bounded [9], in agreement with the experiment [30,31,33–35] an other theoretical studies [36]. Although the transformation performed in this study greatly simplifies the analysis of the system of Eqs (4) and (5), with respect to those of Eqs (1) and (2), another merit of the transformed model is that this transformation does not change the dynamic regimes nor nature of tumor-host interaction. Furthermore, although the parameters *c*, *d* and *f* are dimensionless in Eqs (4) and (5), these three parameters depends on all the biologically meaningful original parameters ($r_{x},K_{x},\alpha_{xy},r_{y},K_{y}$ and $\alpha_{yx}$ in Eqs (3) and (4)). Therefore, the parameters *c*, *d* and *f* sense the changes in six original parameters.

The clinical interpretation of these four dynamic regimes and their natural tumor responses associated depend on degree of immunocompetence (immunocompetent or immunodeficient) of the host, the tumor histological variety, and endogenous perturbations inherent in the cancer and host. In this case, favorable endogenous perturbations may behave as natural anticancer therapies directed at the host, so as to induce metabolic, energetic and physiological conditions unfavorable to the cancer. This may lead to its natural tumor complete remission or natural tumor stationary partial response. If we would have a good understanding of these aspects, the cancer cure would be physiological (withouth the need fon anticancer therapies), idea that should not be underestimate. This would imply that scientific thinking should be directed to the cancer patient as a whole and not to the cancer itself.

### About the existence of periodic orbit

The criterion of Dulac [37,38] states that if the function *R*(*x*,*y*) is defined on a simply connected region $S\subseteq {\mathbb{R}}^{2}$ and the expression $\Gamma =\frac{\partial (PR)}{\partial x}\;+\;\frac{\partial (QR)}{\partial y}$ is not identical to zero and does not change sign, then the system of equations


$$\begin{array}{c} \dot{x}=P(x,y),\\ \dot{y}=Q(x,y), \end{array}$$

has no closed orbits lying entirely in *S*. By taking the function of Dulac $R=u^{m}v^{n}$ [36], m=(cf+f−2)/(1−cf) and n=(cf+c−2)/(1−cf), we get


$$\Gamma =x^{m}y^{n}\frac{cd-1\;+\;f-d}{1-cd}.$$

If cd−1≠d−f, then Γ=0 only when *x* = 0 or *y* = 0. For Regimes II and III, we have that (cd−1+f−d)/(1−cf)<0, then the function Γ do not change its sign inside of any of the quadrants (|u|,|v|>0).

For Regimes I and IV, the condition Γ=0 is fulfilled when cd−1=d−f. In such case, the fixed point *P*_4_ has purely imaginary eigenvalues when *cf* > 1, given by


$$\lambda =\pm i\sqrt{\frac{\left(cd-1)(d-f\right)}{\left(cf-1\right)}}.$$

Since there are not periodic solution in the first quadrant and this system is constrained to nonnegative values of the variables (u,v)≥0, there are not periodic solutions for this model.

## Cancer and host cell populations under an external perturbation

Although the four regimens for dynamic unperturbed tumors reported in “The model and linear stability analysis” section have been reported previously by several authors [7–9], we do not know how these regimes change when the host, not the tumor, is perturbed with external perturbation (e.g., anticancer therapies) and their possible correspondence with results reported in clinical and experimental oncology, as in “Cancer and host cell populations under an external perturbation” section. Furthermore, the clinical interpretation of the four dynamic regimes and types of natural tumor responses should not be confused with those reported when external perturbations, as anticancer therapies are used.

It is well known that the application of oncospecific anticancer therapies (e.g., chemotherapy and radiotherapy) damages both the cancer and the host (specifically the healthy tissue surrounding the tumor) [9,39,40]. In this case, Eqs (3) and (4) can be modified, so that the model includes these therapies, by

$$\begin{array}{c} \dot{x}=r_{x}x(1-\frac{x}{K_{x}})-\alpha_{xy}xy-D(t)\beta_{x}(t,x,y),\\ \dot{y}=r_{y}y(1-\frac{y}{K_{y}})-\alpha_{yx}yx-D(t)\beta_{y}(t,x,y), \end{array}$$ (10)

where *D*(*t*) is the relative dose (dimensionless parameter), which is defined as the ratio between the dose applied to the host with cancer and the maximum permissible dose that the host can receive for the anticancer therapy used. We assume that the effect that this therapy produces in both systems is proportional to *D*(*t*), destroying $D(t)\beta_{x}(t,x,y)$ cancer cells and $D(t)\beta_{y}(t,x,y)$ normal cells per unit of time. The $\beta_{x}(t,x,y)$ and $\beta_{y}(t,x,y)$ coefficients are given in cells/days.

With this model, a crutial point is to fix the functions $\beta_{x}(t,x,y)$ and $\beta_{y}(t,x,y)$ in such a way that the solutions reproduce properly the dynamics of the tumor-host system (an interesting analysis of the relation between a model and their biological counterparts is given in [24] where the correctness of the prey-predator model for tumor-immune interactions is studied). We have considered here the expressions for the functions $\beta_{x}(t,x,y)$ and $\beta_{y}(t,x,y)$ and the model itself as simple as possible to simplify the analysis. More realistic expressions, such as those in [25] and [26], will be object of future research.

We will say that a therapy is applied continuously, or in a constant way (constant external perturbation) if D(t)=D is constant. This corresponds for example to the administration of a medicine in such a way that its concentration in blood is constant along time. It also corresponds to a constant electromagnetic or electric fields applied uninterruptedly to the patient.

We will say that a therapy is applied periodically (periodic external perturbation), if *D*(*t*) is a periodic function of *t*. This corresponds for example to the administration of a drug every certain constant time. Its concentration in blood grows up to a peak, stays in the maximum level for some time and decreases until the medicine is again appliede. The concentration of the farmacus in blood is a periodic function of time. A periodic electromagnetic or electric field applied to the patient would also be a periodic external perturbation.

We will say that a treatment has a constant effect if $\beta_{x}(t,x,y),\beta_{y}(t,x,y)$ are constant. This means for example that the treatment destroys a constant number of cells per unit of time, independently of the size of the existing populations. On the other side, we will say that a treatment has a proportional effect if $\beta_{x}(t,x,y),\beta_{y}(t,x,y)$ are linear functions of *x*,*y*. This means for example that the treatment destroys per unit of time a fraction of the existing cells. The more cells are, the more are destroyed. Other possibilities for the functions $\beta_{x}(t,x,y),\beta_{y}(t,x,y)$ can be considered (see e.g. [25]), but we are not studying them here.

The transformed system of Eqs (6) and (7) becomes in

$$\dot{u}=u-u^{2}-cuv-A_{u},$$ (11)

$$\dot{v}=dv-v^{2}-fuv-A_{v},$$ (12)

with


$$\begin{array}{c} A_{u}=\frac{D}{K_{x}}\beta_{x}(\frac{\tau }{r_{x}},K_{x}u,\frac{r_{x}K_{y}}{r_{y}}v),\\ A_{v}=\frac{Dr_{y}}{r_{x}K_{y}}\beta_{y}(\frac{\tau }{r_{x}},K_{x}u,,\frac{r_{x}K_{y}}{r_{y}}v). \end{array}$$

In the case that the effect of the therapy on the tumor cells does not depend on the healthy cells, that is $\beta_{x}=\beta_{x}(t,x)$, from Eq (11), we can write

$$v=\frac{1}{cu}\left[u-u^{2}-\dot{u}-A_{u}(t,u)\right].$$ (13)

By inserting Eq (13) into Eq (12), it is obtained that

$$\ddot{u}=-\frac{dV}{du}\;+\;\frac{(c\;+\;1){\dot{u}}^{2}}{cu}\;+\;\frac{cd-2\;+\;(2-c-cf)u}{c}\dot{u}\;+\frac{A_{u}^{2}}{cu}+\frac{(2-cd)u+(cf-2)u^{2}-(c+2)\dot{u}u}{cu}A_{u}\;+cuA_{v}+\frac{\text{d}A_{u}}{dt},$$ (14)

where

$$-\frac{dV}{du}=\left(\frac{1}{c}-d\right)u+\left(d+f-\frac{2}{c}\right)u^{2}+\left(\frac{1}{c}-f\right)u^{3}.$$ (15)

Eqs (14) and (15) may be interpreted as the motion of a particle in a viscous medium under the action of a force field [27,28]. In the case that $A_{u}(t,u)=A_{v}(t,v)=0$, the equilibrium points are determined by the potential *V*(*u*). The potential function has three equilibrium points that are determined from the equation $dV(u^{*})/du=0$: ${u_{1}}^{*}=0,{u_{2}}^{*}=1,{u_{3}}^{*}=(1\;-\;cd)/(1\;-\;cf)$. The condition of stability is given by

$$\frac{d^{2}V}{du^{2}}\;>\;0.$$ (16)

**Fig 2:**
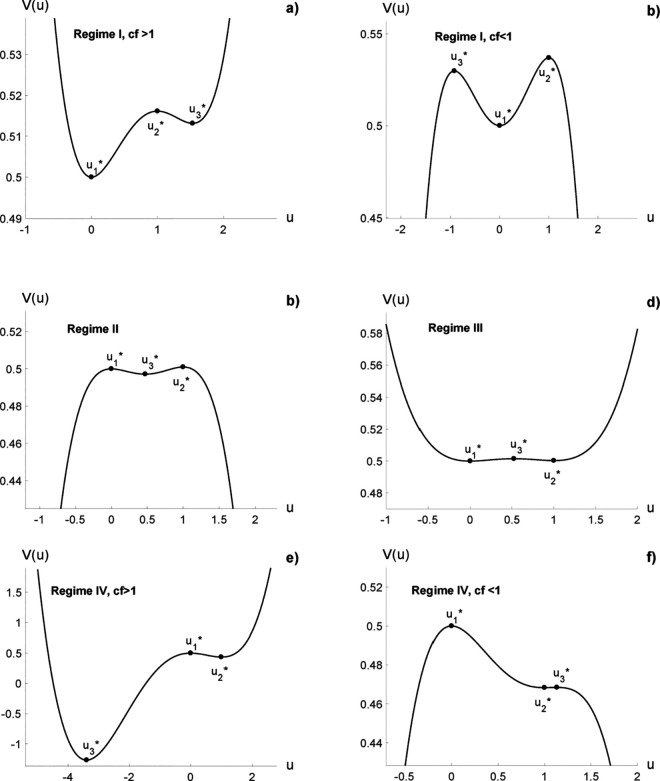
The potential function V(u): a) cd > 1, f < d and cf < 1. b) *cd* > 1, *f* < *d* and *cf* > 1. c) *cd* < 1 and *f* < *d*. d) *cd* > 1 and *f* > *d*. e) *cd* < 1, *f* > *d* and *cf* > 1. f) *cd* < 1, *f* > *d* and *cf* < 1. The value of each parameter is the same used in Fig 1 for b), c), d) and f) subfigures. Subfigure a) was obtained with *c* = 1.65, *d* = 0.75 and *f* = 0.7. Subfigure e) was obtained with *c* = 1, *d* = 0.66 and *f* = 1.1.

By evaluating Eq (16), we find that ${u_{1}}^{*}=0$ is stable for *cd* > 1; ${u_{2}}^{*}=1$ is stable for *f* > *d*; ${u_{3}}^{*}=(1-cd)/(1-cf)$ is stable for *f* < *d*. Note that *V*(*u*) is a polinomial of fourth degree that has three real exrema, two minima and one maximum if *cf* > 1 or two maxima and one minimum if *cf* < 1. There are four possible distinctive cases for the potential depending on the values of the parameters *c*, *d* and *f* (Fig 2).

- **Case 1.:** *cd* > 1 and *f* < *d*
  – If *cf* > 1, ${u_{1}}^{*}$ and ${u_{3}}^{*}$ are minima, ${u_{2}}^{*}$ is a maximum (Fig 2a).
  – If *cf* < 1, ${u_{2}}^{*}$ is a minimum, ${u_{2}}^{*}$ and ${u_{3}}^{*}$ are maxima, (Fig 2b).
- **Case 2.;** *cd* < 1 and *f* < *d* (*cf* < 1)
  – ${u_{3}}^{*}$ is a minimum, ${u_{1}}^{*}$ and ${u_{2}}^{*}$ are maxima (Fig 2c).
- **Case 3:** *cd* > 1 and *f* > *d* (*cf* > 1)
  – ${u_{1}}^{*}$ and ${u_{2}}^{*}$ are minima, ${u_{3}}^{*}$ is a maximum (Fig 2d).
- **Case 4:** *cd* < 1 and *f* < *d*
  – If *cf* < 1, ${u_{2}}^{*}$ is a minimum, ${u_{1}}^{*}$ and ${u_{3}}^{*}$ are maxima, (Fig 2e).
  – If *cf* > 1, ${u_{2}}^{*}$ and ${u_{3}}^{*}$ are minima, ${u_{1}}^{*}$ is a maximum (Fig 2f).

Notice that the Cases 1, 2, 3 and 4 correspond to Regimes I, II, III and IV respectively. Unlike the four regimens for unperturbed tumors (see “The model and linear stability analysis” section), the four regimens shown in this section are for tumors under an external perturbation. These new regimes depend on the performance status of the cancer patient, tumor histological variety and doses *D* of therapy applied to both the cancer and host.

The LV model and the particle-like Eq (14) do not have periodic solutions; nevertheless, the particle may oscillate around the stable equilibrium positions under a periodic driving force. It is possible to define a characteristic or proper frequency around the minima of *V*(*u*) as

$$\Omega^{2}\equiv \frac{d^{2}V(u)}{du^{2}}.$$ (17)

In the case of Regime IV, we have that $\Omega^{2}=f-d$ for $u^{*}=1$.

The behavior of periodic forced nonlinear systems is well known for low dimensional systems [41,42]. The motion is synchronized to the external force for frequencies *ω* of the external force close to Ω [41]. In general, the amplitude of the oscillation decreases as the difference ξ=|Ω−ω| increases and the synchronization is destroyed for higher values of ξ, resulting in a more complex motion that in some cases may be chaotic.

## Cancer and host cell populations under a constant external perturbation

Chemotherapy and radiotherapy cannot be applied continuously over time because of their marked adverse events [9,39,40]. Not so for immunotherapy that is applied to the cancer patient throughout life [43–45]. Nevertheless, several studies report immune-related adverse events that may harm the host. The adverse events induced by immunotherapy in the host are less than those induced by chemotherapy or radiotherapy [46–48].

It is important to note that destruction of healthy host cells is negligible compared to that induced in the tumor, so $\beta_{x}(t,x,y)\;>>\;\beta_{y}(t,x,y)$. Otherwise, constant external perturbation cannot be used because it would induce important adverse events in the host, including death. It is well know that safety and adverse events, in addition to efficacy/effectiveness, are very important therapy aspects aimed at any type of disease must fulfill simultaneously, specifically anticancer therapies.

### Cancer and host cell populations under a constant external perturbation with proportional effect

We assume that the constant external perturbation induces destruction of cancer cells and host cells at constant rate (same amount of cells per unit of time), as long as $\beta_{x}(t,x,y)\;>>$ $\beta_{y}(t,x,y)$. In this case, $\beta_{x}(t,x,y)$ and $\beta_{y}(t,x,y)$ do not depend on time *t*, that is, $\beta_{x}(t,x,y)=\beta_{x}x$ and $\beta_{y}(t,x,y)=\beta_{y}y$. In such case, the LV Eqs (11) and (12) reduce to

$$\begin{array}{c} \dot{x}=r_{x}x(1-\frac{x}{K_{x}})-\alpha_{xy}xy-D\beta_{x}x,\\ \dot{y}=r_{y}y(1-\frac{y}{K_{y}})-\alpha_{yx}yx-D\beta_{y}y, \end{array}$$ (18)

This is again a LV system with growth factors $r_{x}\;-\;D\beta_{x},r_{y}\;-\;D\beta_{y}$ and carrying capacities $K_{x}(r_{x}-D\beta_{x})/r_{x},K_{y}(r_{y}-D\beta_{y})/r_{y}$.

If $r_{x}\;-\;D\beta_{x}\leq 0$, then $\dot{x}\;<\;0$. This implies that *x* is stricttly decreasing and x→0 with time. The constant external perturbation destroys the tumor completely. Therefore, the tumor will be destroyed for highly enough dose *D*. Unfortunately, high dose may destroy also many healthy cells. Consequently, adverse events are induced in the host that may even be severe and subsequently lead to its death.

If $r_{y}\;-\;D\beta_{x}\leq 0$, then $\dot{y}\;<\;0$ and y→0 with time. The constant external perturbation destroys the healthy cells completely. This will not be a problem if the destroyed cells restrict to a limited, non essential, area of the body (e.g., healthy tissue surrounding the tumor), as induced by electrochemical ablation [30,31,33–35].

Let us assume that $D\;<\;\min(r_{x}/\beta_{x},r_{y}/\beta_{y})$. Since $r_{x}\;-\;D\beta_{x}\;>\;0$ and $r_{y}\;-\;D\beta_{x}\;>\;0$, the equations can be transformed to a system of Eqs (6), (7) with coefficients


$$\begin{array}{c} c^{*}=\frac{K_{y}\alpha_{xy}}{r_{y}}=c,\;f^{*}=\frac{K_{x}\alpha_{yx}}{r_{x}}=f,\\ d^{*}=\frac{r_{y}-D\beta_{y}}{r_{x}-D\beta_{x}}, \end{array}$$

and with the transformed variables defined by


$$\begin{array}{c} \tau =(r_{x}-D\beta_{x})t,\;x=(r_{x}-D\beta_{x})K_{x}u,\\ y=(r_{x}-D\beta_{x})K_{y}v. \end{array}$$

Let us analyze the condicions in which the constant external perturbation moves the system to a Regime that takes the cancer patient to a cured state (cancer patient behaves like an apparently healthy individual). We assume that the system is in Regime IV (*cd* < 1 and *d* < *f*) or in Regime III with inadequate initial conditions (*cf* > 1, *cd* > 1 and *f* > *d*).

We wish to move the system to Regime I, Regime II, or even to Regime III for the case of suitable initial conditions (the cancer patient state under external perturbation application). Note that a variation in the dose *D* only affects the coefficient *d* . Then, the constant external perturbation is effective only if *d*  is an increasing function of *D* and the first derivative of *d*  with respect to *D* must be positive ($r_{y}\beta_{x}-r_{x}\beta_{y}\;>\;0$ or $\beta_{x}/\beta_{y}\;>\;r_{x}/r_{y}$).

As usually $r_{x}\gg r_{y}$, $\beta_{x}\ll \beta_{y}$. This means in the therapeutic order that the effect of the constant external perturbation on tumor cells must be much larger than the effect on healthy cells in a proportion at least as the proportion between the growth ratio of the tumor cells over the growth ratio of the healthy cells.

- Cancer patient in Regime IV (*cd* < 1 and *f* > *d*)
  Condition *cd* < 1 is equivalent to $r_{x}\;>\;K_{y}\alpha_{xy}$ and condition *f* > *d* is equivalent to $r_{y}\;<\;K_{x}\alpha_{yx}$. This implies that
  $$\begin{array}{c} r_{y}\beta_{x}-\beta_{y}K_{y}\alpha_{xy}\;>\;r_{y}\beta_{x}-r_{x}\beta_{y}\;>\;0,\\ \beta_{x}K_{x}\alpha_{yx}-r_{x}\beta_{y}\;>\;r_{y}\beta_{x}-r_{x}\beta_{y}\;>\;0. \end{array}$$
  In this situation,
  $$cd^{*}\;>\;1⟺D\geq D_{1}\equiv \frac{r_{y}(r_{x}-K_{y}\alpha_{xy})}{r_{y}\beta_{x}-\beta_{y}K_{y}\alpha_{xy}},$$
  and
  $$d^{*}\;>\;f⟺D\geq D_{2}\equiv \frac{r_{x}(K_{x}\alpha_{yx}-r_{y})}{\beta_{x}K_{x}\alpha_{yx}-r_{x}\beta_{y}},$$
  then,
  – If $D\;>\;\max(r_{x}/\beta_{x},r_{y}/\beta_{y})$, tumor cells and healthy cells go to zero.
  – If $D\;>\;\max(D_{1},D_{2})$, the system goes to Regime I.
  – If *cf* < 1 and $D_{1}\;>\;D\;>\;D_{2}$, the system goes to Regime II.
  – If *cf* > 1 and $D_{2}\;>\;D\;>\;D_{1}$, the system goes to Regime III.
  – If $D\;<\;\min(D_{1},D2)$, the system stays in Regime IV.
  In all the cases, if the constant external perturbation is withdrawn, the system returns to Regime IV, unless the tumor has been completely destroyed. Note that in the mathematical model this is achieved only when t→∞. Nevertheless, in practice this may be accomplished if the variable *x* is smaller than a small quantity.
- Cancer patient in Regime III (*cf* > 1, *cd* > 1 and *f* > *d*)
  Since *cd* > 1 and *d*  is an increasing function of *D*, the condition *cd*  > 1 is satisfied for any D≥0. The condition *f* > *d* is equivalent to $r_{y}\;<\;K_{x}\alpha_{yx}$ and this implies that
  $$\beta_{x}K_{x}\alpha_{yx}-r_{x}\beta_{y}\;>\;r_{y}\beta_{x}-r_{x}\beta_{y}\;>\;0.$$
  In this case,
  – If $D\;>\;\max(r_{x}/\beta_{x},r_{y}/\beta_{y})$, tumor cells and healthy cells go to zero.
  – If $D\;>\;D_{2}$, the system goes to Regime I.
  – If $D\;<\;D_{2}$, the system stays in Regime III.
  The system returns to Regime III when the application of the external perturbation is finished, unless the values of *x*,*y* at this moment are in the region of the plane (*x*,*y*) in which the solution of system of Eqs (6), (7) tends to the equilibium point *P*_2_, that is, if *x* is small enough and *y* is large enough.
- Cancer patient in Regime II (*cf* < 1, *cd* < 1 and *f* < *d*)
  Since *d* > *f*, the condition *cd*  > 1 is satisfied for any D≥0. Condition *cd* < 1 is equivalent to $r_{x}\;>\;K_{y}\alpha_{xy}$ and this implies that
  $$r_{y}\beta_{x}-\beta_{y}K_{y}\alpha_{xy}\;>\;r_{y}\beta_{x}-r_{x}\beta_{y}\;>\;0.$$
  In this case
  – If $D\;>\;D_{1}$, the system goes to Regime I.
  – If $D\;<\;D_{1}$, the system stays in Regime II.
  The system returns to Regime II when the application of the external perturbation is finished, unless the values of *x* at this moment are small enough to be considered zero, that is, the tumor has been destroyed.

Nowadays, anticancer therapeutic strategies has been directed primarily at cancer with host damages, which may be mild, moderate or severe depending on the type of therapy applied [9,39,40,43–49]. Nevertheless, we are not aware that anticancer therapeutic strategies are directed solely at the host in order to create unfavorable conditions in it that lead to complete remission or stationary partial remission of the cancer without adverse events in the host or if these are induced in the host, they are minimal (the host recovers in the shortest possible time). This may be achieved with a constant (see “Host cells population under a constant external perturbation with constant effect” subsection) or periodic (see “Host cells population under a periodic external perturbation with constant effect” subsection) external perturbation.

### Host cells population under a constant external perturbation with constant effect

If we assume that the constant external perturbation destroys a constant number of tumor cells and host cells per unit of time, the system of Eq (10), can be rewritten as

$$\begin{array}{c} \dot{x}=r_{x}x(1-\frac{x}{K_{x}})-\alpha_{xy}xy-D\beta_{x},\\ \dot{y}=r_{y}y(1-\frac{y}{K_{y}})-\alpha_{yx}yx-D\beta_{y}, \end{array}$$ (19)

As the intention of this subsection is to analyze the system when the host is only perturbed with a constant external perturbation, the case considered is the following: $\beta_{x}=0$ and $\beta_{y}\;<\;0$. The effect of the constant term is studied by Kim [23] to explain the effect of harvesting on the dynamic of two competing species. For small values of *A*, the system only undergoes a slight distortion in the phase portrait with a minuscule shift in the equilibrium points. Using the change of variable (Eq (5)) and the change of parameters (Eq (8)), the Eq (19) transforms to


$$\begin{array}{c} \dot{u}=u-u^{2}-cuv,\\ \dot{v}=dv-v^{2}-fuv+A, \end{array}$$

with $A=-D\beta_{y}\frac{r_{y}}{r_{x}^{2}K_{y}}\;>\;0$.

As in the case of previous equations, if the initial conditions *u*(0), *v*(0) are non negative, the solution stays in the positive quadrant, as expected in clinics since no negative populations can happen. Then, only the positive quadrant is considered.

For this system of Eqs (25) and (26), we find the following equilibrium points

$$\begin{array}{c} Q_{1}=\left(0,\frac{d}{2}-\frac{1}{2}\sqrt{d^{2}+4A}\right),\\ Q_{2}=\left(0,\frac{d}{2}+\frac{1}{2}\sqrt{d^{2}+4A}\right),\\ Q_{3}=\left(1-c\frac{f-d-\Delta }{2(cf-1)},\frac{f-d-\Delta }{2(cf-1)}\right),\\ Q_{4}=\left(1-c\frac{f-d+\Delta }{2(cf-1)},\frac{f-d+\Delta }{2(cf-1)}\right), \end{array}$$ (20)

where $\Delta =\sqrt{\left(f-d{)}^{2}-4A(cf-1\right)}$.

**Fig 3:**
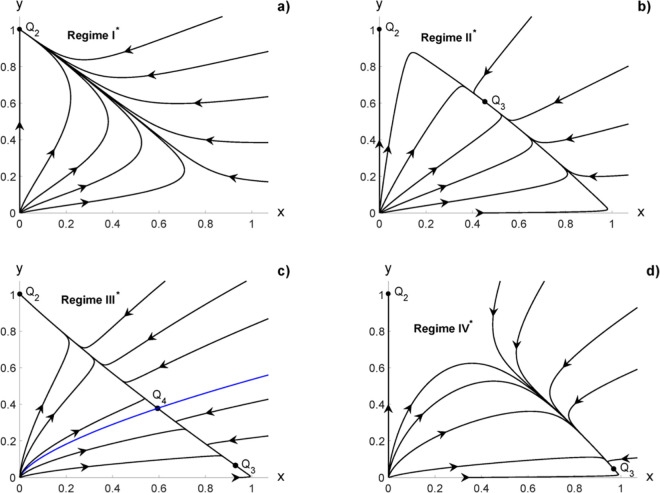
Effect of external stimulus with low continuous dose, in the original coordinates (19): a) Regime I. b) Regime II. c) Regime III. The separatrix is given in blue. d) Regime IV.

For small values of *A*, the fix points are moved slightly with respect to those corresponding to the unperturbed tumor case (Fig 1), as shown in Fig 3. An important fact is that the point (*x*,0) is never an equilibrium point. The population of healthy cells cannot vanish. The Regimes ${IV}^{*}$ is equivalent to Regime II , in which both cancer and healthy cells coexist in the equilibrium point. The size of the equilibrium populations depend on the initial state (Regime II or Regime IV) and on the applied therapy dose.

The dislocation can be considerable and bifurcations can occur for higher values of *A*, as we will expose below.

The point *Q*_1_ has eigenvalues $\lambda_{1}=\sqrt{4A\;+\;d^{2}}\;>\;0$ and $\lambda_{2}=1\;+\;c(-d\;+\;\sqrt{4A\;+\;d^{2}})/2\;>\;0$ and is always unstable. The point *Q*_2_ has eigenvalues $\lambda_{1}=-\sqrt{4A\;+\;d^{2}}\;<\;0$ and $\lambda_{2}=1-c(d\;+\;\sqrt{4A\;+\;d^{2}})/2$ and is stable for $A\;>\;A_{1}$ with

$$A_{1}=\frac{1-cd}{c^{2}}.$$ (21)

In terms of the original parameters, it is stable when

$$D\;>\;D_{1}=\frac{r_{x}r_{y}(r_{x}-K_{y}\alpha_{xy})}{K_{y}\alpha_{xy}^{2}|\beta_{y}|}.$$ (22)

In order for the fixed points *Q*_3_ and *Q*_4_ to exist, the expression inside square root of Δ must be nonnegative. Since *A* > 0, this is always true if cf≤1, as happens in Regime II and also if *cf* > 1 and $A\leq A_{2}$ with

$$A_{2}=(f-d{)}^{2}/4(cf-1).$$ (23)

In terms of the original parameters, this happens when

$$D\;>\;D_{2}=\frac{r_{x}K_{y}(r_{y}-K_{x}\alpha_{yx}{)}^{2}}{4|\beta_{y}|(K_{y}K_{x}\alpha_{xy}\alpha_{yx}-r_{x}r_{y})}.$$ (24)

Moreover, if (*f*–*d*)(*cf*–1) < 0, since Δ≤|f−d|, the points *Q*_3_ and *Q*_4_ have the second component negative and have no interest from the clinical point of view. The equilibrium points *Q*_3_ and *Q*_4_ make sense only when $A\leq A_{1}$, *cf* > 1 and *d* > *f* or well when *cf* < 1 and *d* > *f*. As $A\to A_{1}$, the points *Q*_3_ and *Q*_4_ approach until they collide when *A* = *A*_1_ creating a new equilibrium point, which has the eigenvalues $\lambda_{1}=0$ and $\lambda_{2}=\frac{2-c(d\;+\;f)\;+\;cf(d-f)}{2(cf-1)}\;<\;0$. The point $\tilde{P}$ disappears for higher values of *A*.

- Cancer patient in Regime IV (*cd* < 1 and *f* > *d*)
  In Regime IV and for *cf* > 1, a bifurcation occurs when *A* = *A*_2_ (*D* = *D*_2_), for which *Q*_3_ and *Q*_4_ collide. If $A\;>\;A_{2}$ ($D\;>\;D_{2}$) there are only two equilibrium points: *Q*_1_ is an unstable node whereas *Q*_2_ is stable (it can be verified that $A_{1}\;<\;A_{2}$ in this case) and the patient goes to a Regime I . If $A_{1}\;<\;A\;<\;A_{2}$ ($D_{1}\;<\;D\;<\;D_{2}$) there are two possibilities: If *c*(*f*–*d*) > 2(*cf*–1), the first component of *Q*_4_ and *Q*_3_ are negative, and the patient goes to a Regime I . If *c*(*f*–*d*) < 2(*cf*–1), *Q*_3_ and *Q*_4_ are in the positive quadrant, and the patient goes to Regime III . Finally, if $A\;<\;A_{1}$ (*Q*_2_ and *Q*_4_ collide at *A* = *A*_1_), the point *Q*_2_ is not stable but *Q*_3_ is, and the patient goes to Regime II .
  When *cf* < 1 on regime IV, the second component of point *Q*_4_ is negative and this point has no interest from the biological point of view. Moreover, a bifurcation occurs when *Q*_2_ and *Q*_3_ collide, which happens when *A* = *A*_1_. For $A\;>\;A_{1}$ ($D\;>\;D_{1}$), the first component of *Q*_3_ is negative and there are only two equilibrium points: *Q*_1_ is an unstable node whereas *Q*_2_ is stable and the patient goes to Regime I . If $A\;<\;A_{1}$, *Q*_2_ is not stable but *Q*_3_ is stable. The patient goes to Regime II .
  In conclusion,
  – If $D\;>\;\max(D_{1},D_{2})$, the system goes to Regime I .
  – If *cf* < 1 and $D\;<\;D_{1}$ ($D_{2}\;<\;0$), the system goes to Regime II .
  – If *cf* > 1, *c*(*f*–*d*) > 2(*cf*–1) and $D\;>\;D_{1}$, the system goes to Regime I .
  – If *cf* > 1, *c*(*f*–*d*) < 2(*cf*–1) and $D_{2}\;>\;D\;>\;D_{1}$, the system goes to Regime III .
  – If $D\;<\;D_{1}$, the system goes to Regime II .
- Cancer patient in Regime III (*cd* > 1, *cf* > 1 and *f* > *d*)
  In this regime, $A_{1}\;<\;0$, therefore, *Q*_2_ is always stable. If $A\;<\;A_{2}$, *Q*_3_ and *Q*_4_ are in the positive quadrant, *Q*_4_ is not stable and *Q*_3_ is stable. The patient goes to Regime III . If $A\;>\;A_{2}$, the patient goes to Regime I .
  In conclusion,
  – If $D\;>\;D_{2}$, the system goes to Regime I .
  – If $D\;<\;D_{2}$, the system goes to Regime III .
- Cancer patient in Regime II (*cd*  <  1, *cf* < 1 and *f* < *d*) In this regime, *Q*_4_ is never in the positive quadrant. If $A\;<\;A_{1}$, *Q*_3_ is stable but *Q*_2_ is not, and the patient goes to Regime II . If $A\;>\;A_{1}$, *Q*_2_ is stable and *Q*_3_ has the first component negative. The patient goes to Regime I .
  In conclusion,
  – If $D\;>\;D_{1}$, the system goes to Regime I .
  – If $D\;<\;D_{1}$, the system goes to Regime II .

The results of this subsection demonstrate that it is possible to obtain complete tumor remission for sufficiently high doses of a constant external perturbation. This is impressive because a very aggressive tumor may be destroyed not by direct attacking it but enhancing the host (e.g., stimulating the immune system) using high constant doses of an external perturbation. Unfortunately, such powerful stimulating anticancer therapies are not available at present due to the possible induction of adverse events in the host. This is true for currently existing anticancer therapies.

Although a constant external perturbation over a host may induce complete tumor remission, its exposure time may be short or long depending on whether the adverse events in the host are marked (irreversible tissue damage or death) or not. If adverse events are marked, the exposure time of this constant external perturbation is of short duration; otherwise, it is of long duration.

In this study, high dose is referred to that applied to the host in such a away as to sufficiently potentiate the immune system and/or create unfavorable conditions in the host in order to induce complete remission or stationary partial response of the tumor through the apoptosis mechanism without provoking severe adverse events in the whole organism. This may suggest the tumor sensitivity to the external perturbation applied to the host must be much greater than that of the rest of the organism. In other words, the tissue damage induced in the tumor is amplified several orders of magnitude compared to that of the rest of the organism. This would represent a new concept for cancer therapy, unprecedented in the literature.

In the therapeutic order, the external constant perturbation may confirm that the target of anticancer therapies should be the different types of cells that compose the tumor stroma, rather than the cancer cells, for the long-term control of this disease [50–52]. In principle, this external perturbation type may be a biological or physical therapy, which may be combined. Among the biological therapies may be mentioned anti-VEGF therapy (antiangiogenic therapy) [53–56], immunotherapy using immune checkpoint inhibitors (anti-chekpoint therapies) [57–59], combination of antibody therapies [60,61]. Nevertheless, the use of these anticancer therapies may induce severe adverse events [62–64]. On the other hand, constant electric stimulation therapies applied directly to the host for restoration of the tumor electrical microenvironment [65–69]. This may lead to modifications of the tumor stroma, either by repolarization of its components, reestablishment of the pH of the environment, inhibition of blood vessels, among others. Electrical therapies should not be confused with those applied directly to the tumor (e.g., electrochemical ablation) [30,31,33–35].

Consequently, these tumor stroma-targeted anticancer therapies need to be redesigned or new therapeutic strategies proposed. A more reasonable therapeutic strategy is the application of an external stimulus applied periodically to host cells population. This may be modelled by taking the applied dose as a (periodic) function of time, or equivalently taking $\beta_{x}$ and $\beta_{y}$ periodical functions of time.

### Host cells population under a periodic external perturbation with constant effect

As a first approximation we will assume that $\beta_{x}(t,x,y)=\beta_{x}$ and $\beta_{y}(t,x,y)=\beta_{y}$ are constant, and that the dose D(t)=D(1−cos(ωt) is applied periodically. In such a case, the LV equations reduce to

$$\dot{x}=r_{x}x(1-\frac{x}{K_{x}})-\alpha_{xy}xy-D(1-\cos(\omega t))\beta_{x},$$ (25)

$$\dot{y}=r_{y}y(1-\frac{y}{K_{y}})-\alpha_{yx}yx-D(1-\cos(\omega t))\beta_{y}.$$ (26)

Moreover, we will consider here the case where the external stimulus applied periodically affects only the host cells, with $\beta_{y}\;<\;0$. Then, the system of Eqs (25), (26), reduces to

$$\dot{x}=r_{x}x(1-\frac{x}{K_{x}})-\alpha_{xy}xy,$$ (27)

$$\dot{y}=r_{y}y(1-\frac{y}{K_{y}})-\alpha_{yx}xy\;+\;D|\beta_{y}|(1-\cos(\omega t)).$$ (28)

**Fig 4:**
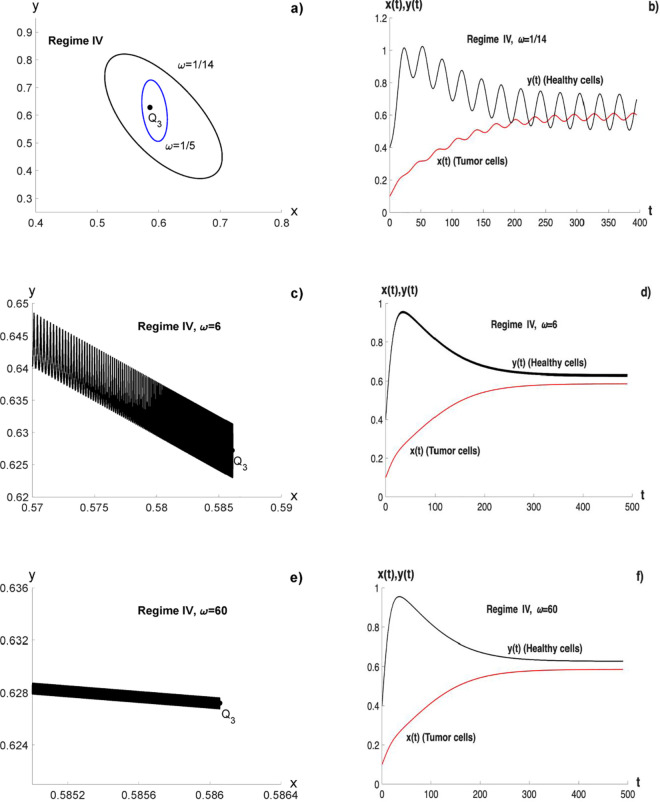
System on regime IV under a periodic perturbation, in original coordinates: a) limit cycle for ω~Ω; b) solutions for ω=1/14; c) limit cycle for ω=6; d) solutions for ω=6; e) non-chaotic attractor for ω≫Ω; f) solutions for ω=60. The parameters values are *c* = 1, *d* = 0.66, *f* = 1.1 and *A*_1_ = 0, which gives Ω=0.2 and *A* = 0.175.

An analytical study is not easy, so simulations are performed by numerically integrating these systems of differential equations for Regime IV. A limit cycle is observed around the position where *Q*_3_ was located (see Fig 4a)). The oscillations of periodically forced systems synchronize to the driving term and the amplitude of oscillations decreases with the increase of the frequency *ω* (these occurs in a certain range of *ω* around the characteristic frequency Ω (Eq (17)) of the unperturbed system) [36,37], as it is shown in Fig 4b) and 4d). For higher values of *ω* the limit cycle becomes becomes very small and in the limit when *ω* tends to infinity, it tends to an attractor (see Fig 4e) and 4f)).

The subfigures in Fig 4 are obtained using the original coordinates, taken as parameters $K_{x}=K_{y}=1$ cells, *r*_*x*_ = 0.1 days^−1^, *r*_*y*_ = 0.066 days^−1^, $\alpha_{xy}=0.066$ cells^−1^days^−1^, $\alpha_{yx}=0.11$ cells^−1^days^−1^, $\beta_{y}=-1/40$ and *D* = 1. This gives *c* = 1, *d* = 0.66 and *f* = 1.1; therefore, *cd* < 1 and *f* > *d* that correspond to Regime IV.

Since the proposed model (27), (28) is equivalent to a second order equation with a periodic external impact and a double-well potential function, it is capable of exhibiting chaos. The study of the absence or presence of chaos in the system is not an easy task, and a reliable conclusion would require a multiparametric bifurcation analysis (see e.g. [70]). Such an analysis is beyond the scope of this paper and can be the subject of additional research. Here we will only study numerically the behaviour of the maximum Lyapunov exponents when we vary the parameters of the system, verifying that they are non negative, showing that the existence of chaos is very unlikely.

We calculate the maximum Lyapunov exponents [38], defined as

$$\sigma \equiv \underset{d(\vec{r_{0}},0)\to 0}{\lim_{t\to \infty }}\frac{1}{t}ln\frac{d(\vec{r},t)}{d(\vec{r_{0}},0)},$$ (29)

where $d\equiv \|\Delta \vec{r}(t)\|$ is the distance between two initially close trajectories, that is, the trajectories where at $\vec{r_{0}}$ and $\vec{r}\;+\;\Delta \vec{r_{0}}$ at *t* = 0. The evolution of distance between both trajectories is calculated from the linearized system

$$\frac{d(\Delta \vec{r})}{dt}=\vec{M}(\vec{r}(t))\cdot \vec{r},$$ (30)

where $\vec{M}(\vec{r}(t))$ is the Jacobi matrix of the system of Eqs (27)–(28) evaluated along a fix trajectory $\vec{r}=\left(x(t),y(t)\right)$. To avoid numerical overflow of the distance $d(\vec{r},t)$, it is chosen a small fixed time interval *τ*, and *d* was renormalized to *d*_0_ every *τ*. Thus, we iteratively compute the values

$$d_{k}=\|\Delta {\vec{r}}_{k-1}(\tau )\|,$$ (31)

$$\Delta \vec{r_{k}}(0)=\frac{\Delta {\vec{r}}_{k-1}(\tau )}{d_{k}},$$ (32)

where $\Delta {\vec{r}}_{k-1}(\tau )$ is obtained by integrating Eq (30), with the initial value $\Delta {\vec{r}}_{k-1}(0)$. Defining the quantity

$$\sigma_{n}=\frac{1}{n\tau }\sum_{i=1}^{n}ln\left(\frac{d_{i}}{d_{0}}\right),$$ (33)

it can be shown that for a *τ* not too large $\lim_{n\to \infty }\sigma_{n}=\sigma$ and is independent of *τ*. The other can be computed in a similar way, but using an ortogonalization process.

**Fig 5:**
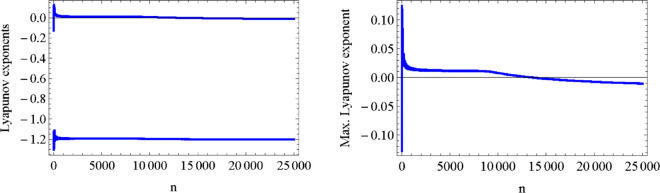
Both Lyapunov exponents as function of the steps n (leftt) and maximun Lyapunov exponent $\sigma_{n}$ (right) as a function of n.

In order to study the behaviour of the values of the Lyapunov Characteristic Exponents (*LCEs* )when varying the parameters, we have searched for local maxima in a region $D\in {\mathbb{R}}^{5}$ of the parameters (c,d,f,A,ω) by means of a Monte-Carlo type method. We have used the Mathematica toolbox developed by Marco Sandri in [71], using τ=0.1, integrating numerically the associated differential equations with time step *h* = 0.02 After a very time consuming search, we have found that the paerameters *c* = 0.933816, *d* = 0.999086, *f* = 1.27836, *A* = 0.100613 and ω=0.496117 yield a local maximum. For these values of the parameters we get a negative maximum Lyapunov exponent, close to zero. Fig 5 shows the behavior of both Lyapunov exponents (left) and the maximun Lyapunov exponent $\sigma_{n}$ (right) as a function of the steps n. The figures show the convergence to negative values.

**Fig 6:**
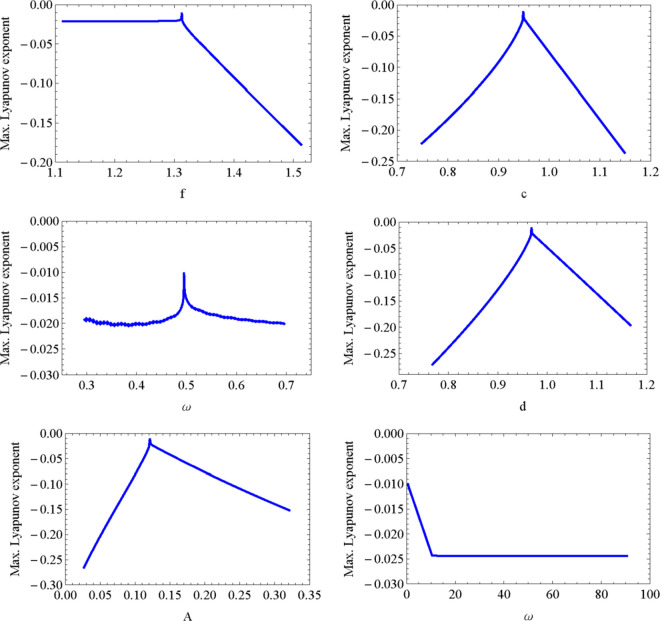
Maximum (Max.) Lyapunov exponent as a function of each of the model parameters c (top left box), d (top right box), f (center left box), A (center right box) and ω (lower left and right boxes).

The dependence of the maximum Lyapunov exponent *σ* on the parameters is shown in Fig 6 where each subfigure shows the value of the exponent against one of the parameters, leaving the other four unchanged. The maximum Lyapunov exponent remains negative in all the cases. The maximum exponet for high frequencies, is shown in the last subfigure of Fig 6. It is observed in the figures a discontinuity in the maximum Lyaponov exponent at the maximum. This may be explained because the maximum happens precisely at a bifurcation point where points *Q*_3_ and *Q*_4_ collapse when Δ becomes zero. Both points become complex and dissapear from the phase space for higher values of the patameter A.

Negative Lyaponov and maximum Lyaponov exponents for all values of the parameters not only confirm stability, convergence and synchronization, but also that the tumor-host system is dissipative (appearance of coherent and self-organized structures and anisotropy in system far from equilibrium, as open system), in agreement with other authors [31,72,73,87,88].

In the therapeutic order, the external periodic perturbation may be of a biological or physical nature that may be combined as well. There exist moderated stimulating biological anticancer therapies whose action are not constant, but may be applied to the patient periodically, such as the use of cytokines and vaccines to enhance the response of the immune system [41,74–78]. Furthermore, physical therapies may be an electromagnetic field at certain frequencies (alternating electric fields and/or a periodically applied pulsed electromagnetic field) that may harmonize the host (restoration of injury and disorders caused by the tumor in its vecinity), modulate the tumor stroma, stimulate the immune system response and inhibits tumor angiogenesis in order to induce the complete remission or stationary partial response (limit cycle: cancer as a controlled chronic disease). For this, the ideas reported in several studies must be taken into account [79–85].

## Limitation of the study

The fundamental limitation of this study is that the theoretical results obtained with the different models have not been experimentally validated; therefore, they have no direct interpretation in clinical oncology, nor do they allow for inferring specific therapeutic interventions. Although *x*(*t*) and *y*(*t*) in the LV model have not been experimentally measured in this study, they can be quantified over time by means of a flow cytometry [86]. Nevertheless, most studies focus on the tumor kinetics (by means of its tumor volume *V*(*t*), tumor mass *M*(*t*), tumor density that changes in space-time ρ(x,t), or population of tumor cells *x*(*t*), not so on that of *y*(*t*) [31,32,34–36,87,88]. Therefore, the partial experimental validation of the LV equation in this study can be done only for *x*(*t*), but not for *y*(*t*).

As *V*(*t*) and *x*(*t*) may be used interchangeably to describe tumor growth kinetics [87,88], *x*(*t*) and *x*_0_ in the LV equation can be replaced by *V*(*t*) and $V_{0}$, respectively. This may be argued because the tumor volume is the most frequently used in experimental oncology [31,32,34–36,87,88] and *x*(*t*) spatially occupies all *V*(*t*), and thus *V*(*t*) is directly proportional to *x*(*t*). In order to validate LV equation (only for *x*(*t*) or *V*(*t*)), we fit unperturbed Ehrlich tumor volumes, growing in BALB/c/Cenp mice, reported in [88]. The results of the fitting with LV equation are compared with those obtained for the Montijano-Bergues-Bory-Gompertz (MBBG) which have been previously used to describe this Ehrlich tumor growth kinetcs [32,88].

Using a minimum squares approach, the parameters α=0.719 days^−1^, β=0.261 days^−1^, $V_{obs}=0.190$ cm^3^ and V(0)=0.500 cm^3^ were obtained in [88] for the MBBG equation. Here, fitting the same data to the LV model we have obtained the parameters *r*_*x*_ = 4.83671566 days^−1^, *r*_*y*_ = 6.36103209 days^−1^, $\alpha_{xy}=1.52999021$ days^−1^/cm^3^, $\alpha_{yx}=1.97864993$ days^−1^/cm^3^ *K*_*x*_ = 3.21508273 cm^3^, *Ky* = 2.78479322 cm^3^, *x*_0_ = 0.46733650 cm^3^, and *y*_0_ = 2.38718513 cm^3^. It is remarkable that $\alpha_{xy}≃r_{x}/K_{x}$ and therefore $\delta_{xy}≃0$ which means that the healthy cells do not practically destroy tumor cells.

**Fig 7:**
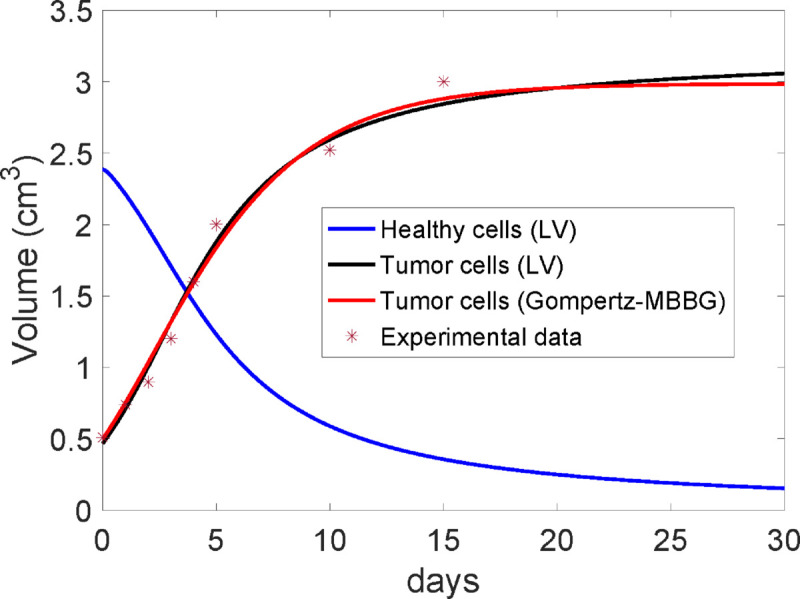
Fitting of the evolution of the unperturbed Ehrlich tumor volume given by the Lotka-Volterra and Gompertz-MBBG models to experimental data. Star points correspond to the experimental data, black line to the LV model and red line to the MBBG equation. Blue line corresponds to the evolution of the volume of healthy cells given by the LV model.

In Fig 7, we plot the tumor volume (in cm^3^) versus time (in days) for the experimental unperturbed Ehrlich tumor volumes (star points), LV equation (black line), and MBBG equation (red line). Although *y*(*t*) is not fitted to experimental data, the theoretical behavior of *y*(*t*) for LV equation (blue line) is also shown in Fig 7. From these results, the proposed LV equation (only for *x*(*t*) or *V*(*t*)) adequately describes the unperturbed Ehrlich tumor volumes and agrees with MBBG equation, which in turn is consistent with the conventional Gompertz equation [88] and Avrami equations [32,37]. A measure of the correctness of LV model can be given by the Haussdorf distance between the curve *V*(*t*) corresponding to the MBBG equation and the one for the LV model, which is 0.0415. This shows that both models give a very similar approximation to the experimental data.

## Scientific novelty of the study

Despite the main limitation of this study, these results lay the groundwork for searching for existing anticancer therapies or proposing new therapeutic interventions that allow complete remission or prolonged control of larger solid tumors (tumor volumes ≥5 cm^3^) with constant and periodic perturbations directed solely at the host, unprecedented in the literature. This is possible because this new model has the capacity to regulate the dynamics of cell populations in the tumor and provides support for an alternative therapeutic paradigm in cancer. Our results lay the groundwork for proposing future *in silico* studies that allow the inclusion of diverse external perturbations in the model proposed in this study, some targeting the tumor and others targeting the host. This study and its results are consistent with the current trend of combining therapies against cancer cells and tumor stroma. This will allow us to understand the specificity and effectiveness of each of these external perturbations, which can be applied on the same scale or at different times. Furthermore, the predictive capacity of the model proposed in this study can be evaluated. The latter is possible through knowledge of the parameter spaces of our model and these perturbations that allow for long-term cancer control or complete remission.

The results of this study lay also the groundwork for directing the model essentially to one of the challenges of current oncology: the long-term control of advanced tumors (that have already metastasized), which are considered incurable, and anticancer therapies targeting them are applied with palliative rather than curative intent. Furthermore, the results may suggest that counterintuitive hypotheses should be tested in preclinical and clinical studies aimed at the very long-term management of advanced cancer.

## Possible application of periodic external perturbation in experimental oncology

**Fig 8:**
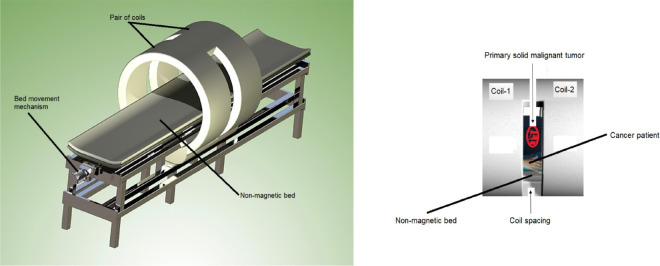
Schematic representation of: a) magnetic bed (left subfigure) and b) cancer patient lying in nonmagnetic bed inside the magnetic system formed by a pair of coils (courtesy of Luis Bergues Cabrales) (right subfigure).

Periodic external perturbations targeting only the host may be implemented in experimental oncology, a novel and unprecedented aspect in the scientific literature. One possible way of applying such host-specific periodic external perturbation may be by means of ultra low frequency electromagnetic fields (ULF-EMF: frequency range (0-30] Hz), extremely low frequency-electromagnetic field (ELF-EMF: frequency range (30-100] Hz) or an extremely low-frequency pulsed electromagnetic field (PEMF: frequencies ≤100 *Hz*) generated by a magnetic bed, as shown schematically in Fig 8. This magnetic bed comprises a magnetic system (e.g., a pair of coils), a suspended non-magnetic bed (e.g., wood) an the associated electronics (e.g., electrical current source).

The coils in this pair have the same inner diameter but different lengths (L1 and L2) and are separated by a distance (D). Electric currents flow through them in opposite directions (I1 = −I2), creating a configuration in which the magnetic induction at each point in the central region of the magnetic system, where the primary tumor is located, is either zero or low enough (≤0.1 *mT*) to avoid any biological effect, such as stimulation or inhibition of the tumor. Ideally, the total length L1 + D + L2 should match the height of the cancer patient. The later can be guaranteed by using two pairs of coils with different lengths, inner diameters and separations between them.

The patient lies on a movable bed that can be adjusted within the magnetic system. This allows that the organism region containing the primary tumor to be positioned at the magnetic center, which coincides with the mechanic center of the system. This ensures that the tumor region does not receive the magnetic induction generated by this magnetic system. The electronics guarantees the waveforms, frequency ranges, among other parameters.

The same idea suggested for cancer patients may be carried in laboratory animals with cancer, but ULF-EMF, ELF-EMF and PEMF are generated by a mini magnetic bed (similar to Fig 8), whose dimensions are on the order of animal species size. In this case, the animal is sedated and securated in a stock to guarantee that the primary tumor is in the center of the magnetic system. Specific details of the spatial map of magnetic field (e.g., presence of linear and higher-order harmonics) for these therapeutic purposes are not provided in this study because they be the claim of a future patent. For this, aspects of electronic, molecular and cellular biology of cancer must be taken into account.

The positioning of the organism region, either a human or experimental animal biomodel, that contains the tumor ensures that ULF-EMF, ELF-EMF and PEMF do not stimulate the cancer angiogenesis process, as report in the scientific literature [89]. This is not favorable for the cancer patients because the tumor angiogenesis is one of cancer hallmarks for its growth, progression and metastasis [90]. The angiogenesis proliferation induced by these three physical therapies may explain, in part, why they do not induce complete remission in most tumor types reported in *in vitro* and *in vivo* studies, but delay cancer cell growth and proliferation by apoptosis induction (caspase pathway) and alterations in the genetic expression of cancer cells, among others findings [91,92]. Furthermore, it is advisable that the entire host, except for the part containing the primary tumor, be inside the coils that make up the magnetic system because ULF-EMF, ELF-EMF and PEMF have anti-inflammatory properties and activate the immune system [93]. The anti-inflammatory properties of these three therapies have been explained by the inhibition of pro-inflammatory cytokines (e.g., IL-6 cytokines) and activation of anti-inflammatory ones (e.g., IL-10 cytokines) [93,94].

It is important to note that the design of the magnetic bed suggested in this study is only valid when there is only one primary malignant tumor in patients or experimental biomodels with cancer. In addition, this procedure may be applied to organims with a larger benign tumor, either superficial or deep. If patients or experimental biomodels with cancer have one or more primary tumors and multiple metastatic nodules, this proposal cannot be used. In this case, ULF-EMF, ELF-EMF and PEMF must be directed in another direction, which is the subjected of a novel patent; therefore, no details are given.

## Summing up and outlook

When the oncoespecific anticancer therapies, immunotherapy and physical therapies are used, the complete remission of highly aggressive tumors is observed less frequently. This also occur for advanced tumor stages. This tumor response type does not always mean the cancer is completely cured, aspect that may be explained because some cancer cells may remain in body for many years after application of these therapies. Furthermore, these therapies reduce the size of these tumors, but they may regrow at a later time after the application of these external perturbations directed to the tumor. In the worst scenario, these therapeutic strategies may accelerate the death of the patient.

The LV equation is used to test two different therapeutic strategies. First, we study the action of a constant external perturbation to healthy cells of the host. In principle, it should be possible to eradicate the malignant cells choosing the appropriate doses and exposure time. Unfortunately, such external perturbations are not available in clinical or experimental oncology at present. This may be due to their doses and exposure time are limited by the tolerance of the organism. For this reason, the redesing of current biological and physical therapies or the conception of new anticancer therapies aimed at the host. In both cases, these therapies have to be safe, induce minimal adverse events in the host, effective and low cost (compared to existing anticancer therapies).

A second strategy is the stimulation of the action of a continuous periodic external perturbation. Under the action of a periodic external perturbation appears a limit cycle, i.e., both populations oscillate and may coexist indefinitely without extinction. This result is predicted very early by ecological model and more recent it is found in the study of dynamical models of the immune system.

It results interesting that for higher frequencies of the periodic external perturbation, the limit cycle becomes a non-chaotic attractor, which reduces its size as the frequency of this perturbation is increased. These results indicate that malignant tumors may be controlled, but not cured. The latter is of great importance and relevance in cancer therapy since this disease may become chronically controlled (stationary partial response [30]). A merit of this study is that it demonstrates that cancer may be controlled through the appropiate application of a periodic external perturbation to the host and not only to the tumor. The same idea of cancer control, instead of its complete cure, has been reported by other authors from the approach of evolutionary ecology [42,95–97].

Undoubtedly, these theoretical results need to be tested in preclinical studies and subsequently in clinical ones. This will allow us to evaluate the validity, feasibility and accuracy of the model proposed in this study, as well as the search for others new appropiate periodic external perturbations to the host. Consequently, the LV model may serve as a guide for the design and interpretation of experiments.

The results obtained for the different values of the frequency for the periodic external perturbation may suggest the possible existence of one or several resonance frequencies that potentiate and harmonize the host that may lead to the host governing indefinitely the tumor-host dynamics or the tumor complete remission. This aspect can be addressed in an additional study, taking into account the results reported in previous studies [98–100].

Finally, it is important to point out that the behavior of the higher dimensional LV model, taking into account the effect of other types of cells, is a problem that require further studies. Not only because they are mathematically interesting, but also because they may be crucial in the control of the cancer. Furthermore, although the intention of this study is to model the tumor-host dynamics when the latter is perturbed with a constant or periodic external perturbation, it lays the foundations for generalizing the model when anticancer therapies directed ath the tumor and others directed at the host are used, as occur in the combination of immunotherapy with chemotherapy and/or radiotherapy [43–45], and immunotherapy with irreversible electroporation [101–104]. According to our results, periodic external perturbations with different parameters may be used to perturb the tumor and host in order to understand how the tumor-host dynamics changes. Additionally, it is important to take into account the circadian immune system [105]. These aspects will be analyzed in a future study.
